# Prognosis and Characterization of Immune Microenvironment in Head and Neck Squamous Cell Carcinoma through a Pyroptosis-Related Signature

**DOI:** 10.1155/2022/1539659

**Published:** 2022-04-06

**Authors:** Lin Lu, Peiling Zhang, Xiaofei Cao, Mingmei Guan

**Affiliations:** Department of Medical Oncology, Guangzhou First People's Hospital, School of Medicine, South China University of Technology, Guangzhou, Guangdong 510180, China

## Abstract

Pyroptosis, as a novel identified programmed cell death, is closely correlated with tumor immunity and shows potential roles in cancer treatment. Discerning a pyroptosis-related gene signature and its correlations with tumor immune microenvironment is critical in head and neck squamous cell carcinoma (HNSCC). Transcriptome data and corresponding clinical data were downloaded from TCGA and GEO databases. Tumor mutation burden (TMB) data were obtained from TCGA database. Firstly, univariate and least absolute shrinkage and selection operator (LASSO) regression analyses were used to construct a six pyroptosis-related gene signature. Kaplan–Meier analysis, receiver operating characteristic (ROC) curves, and principal component analysis (PCA) results verified that the risk model has good performance in predicting the survival. Gene Ontology (GO) and Kyoto Encyclopedia of Genes and Genomes (KEGG) analyses revealed that the pyroptosis-related gene signature was immune related. Finally, the immune landscape and immunotherapy sensitivity prediction capabilities of the risk model were further explored. There were close correlations between the overall survival (OS) and various immune cells and immune functions. Single-sample gene set enrichment analysis (ssGSEA) showed that high risk group had decreased expression of various immune cells and lower activities of immune functions. Meanwhile, tumor mutation burden (TMB) data combining risk score could well predict the OS of HNSCC patients. However, tumor immune dysfunction and exclusion (TIDE) analysis revealed that there was no significant difference in the sensitivity to immunotherapies between high and low risk groups. Finally, a nomogram based on risk score and clinicopathological parameters was constructed. And, the risk model demonstrated better sensitivity and specificity than TIDE scores and T-cell-inflamed signature (TIS). In conclusion, although the risk model could not well predict the immune escape and response to immunotherapies, the signature established by pyroptosis-related genes, with better sensitivity and specificity than TIDE scores and TIS signature, could be used for predicting prognosis and immune status of HNSCC patients.

## 1. Introduction

Head and neck cancer, including tumors originated from oral cavity, nasopharynx, oropharynx, hypopharynx, larynx, and tongue, accounts for more than 830,000 newly diagnosed cases worldwide [1]. Approximately 90% of head and neck cancers are head and neck squamous cell carcinoma (HNSCC). The primary treatment strategies for HNSCC include surgery, radiotherapy, and chemotherapy. The 5-year survival rate is approximately 50–60% due to the high heterogeneity of HNSCC [2, 3]. Recently, immune checkpoint inhibitors (ICIs), such as anti-PD1/PD-L1 and anticytotoxic T lymphocyte antigen 4 (CTLA-4), have been applied in tumor treatment [4]. However, only a small population of patients could respond to PD-1/PD-L1 antibodies [5, 6]. Thus, identifying biomarkers that could be used as prognostic factors and treatment targets is an urgent need in HNSCC.

Pyroptosis, also known as caspase 1-dependent programmed cell death, has gained a lot of attentions recently. Pyroptosis could be triggered by microbial infection and noninfectious stimuli, such as cancers [7]. Pyroptosis is morphologically and mechanistically distinct from other forms of cell death, which is characterized by cell swelling, rapid plasma membrane rupture, and release of proinflammatory cytokines [8, 9]. The biological functions of pyroptosis in the tumor process are still controversial. On the one hand, as a lytic form of programmed cell death, pyroptosis could mediate tumor cell killing [10, 11]. On the other hand, as a form of proinflammatory death, pyroptosis could promote tumor growth by forming a suitable tumor growth microenvironment [12]. Multiple pyroptosis-related genes have been identified in various malignancies, including head and neck cancer. GSDME-mediated pyroptosis promoted the cytotoxicity of triptolide against head and neck cancer cells [13]. NLRP3 inflammasome was involved in pyroptosis activation of HNSCC cells [14]. However, the roles of pyroptosis related genes in predicting the survival of HNSCC patients are still largely unknown.

Chemotherapy and targeted therapy could eliminate tumor cells by inducing the pyroptosis of tumor cells [15, 16]. And induction of pyroptosis has been viewed as a potential cancer treatment strategy [13]. Moreover, pyroptosis could mediate tumor regression by initiating antitumor immunity [17]. However, the therapeutic roles of pyroptosis in HSCC still need to be elucidated.

In this study, we firstly establish a pyroptosis gene signature for the prediction of OS in HSNCC. Secondly, the performance and biological functions of the risk model were further investigated. Moreover, the associations with immune microenvironment and immunotherapy responses were explored. Finally, a nomogram was established to predict the OS of patients with HNSCC.

## 2. Materials and Methods

### 2.1. Data Download and Processing

RNA-sequencing data and corresponding clinical data were downloaded from the TCGA database (https://tcga-data.nci.nih.gov/tcga/) and the NCBI Gene Expression Omnibus (GEO) database. The accession number of GEO data was GSE65858, and the data platform number was GPL10558. TCGA-HNSCC cohort included 44 normal tissues and 502 HNSCC tumor tissues. GSE65858 contained RNA-sequencing data and complete clinical information of 270 HNSCC patients. All data were obtained from online public sources. Thus, ethical approval was not required.

### 2.2. Identification of Pyroptosis-Related Genes

A list of 52 pyroptosis genes was prepared by screening published literatures [17–20]. The expression of pyroptosis genes was retrieved using “limma” package in R software. Differentially expressed pyroptosis-related genes with significant differences were compared between normal tissues and HNSCC tumor tissues with *P* < 0.05. Differentially expressed pyroptosis genes were visualized using a heatmap by utilizing “pheatmap” package in R software.

Protein-protein interaction (PPI) network of these differentially expressed pyroptosis-related genes was constructed using Search Tool for Recurring Instances of Neighbouring Genes (STRING) database (https://string-preview.org/). The correlations between these differentially expressed pyroptosis genes were visualized using “igraph” and “reshape2” packages in R software.

### 2.3. Consensus Clustering Analysis

Patients were classified into different groups based on the best k-means clustering by using the ConsensusClusterPlus R package. Cluster consistency clustering analysis was based on the expression of 6 pyroptosis-associated genes by setting the cluster variables (*k*) set from 2 to 10.

Kaplan–Meier survival analysis between different clusters was performed by using “survival” and “survminer” packages in R software. The correlations of different clusters with clinical parameters (Age, Gender, Grade, Stage, and T, N, and M stages) were assessed and demonstrated by a heatmap using “pheatmap” package.

### 2.4. Univariate Cox Regression Analysis

Pyroptosis genes with prognostic values were identified using univariate cox regression analysis with *P* < 0.05. Least absolute shrinkage and selection operator (LASSO) regression analysis was used to construct a risk model by using “glmnet” and “survival” packages.

### 2.5. Performance of the Risk Model

Firstly, risk score was calculated by the following formula: expression value of gene1∗coefficient of gene1+…+Expression of geneN∗coefficient of geneN. Coefficient of each gene was generated from multivariate Cox regression. HNSCC patients were divided into high risk and low risk group by using the median risk score as a cutoff. The performance of the risk model was evaluated using Kaplan–Meier survival analysis and the area under curve (AUC) of the receiver operating characteristic (ROC) curves. Kaplan–Meier survival analysis was used to compare the survival rate between high risk and low risk groups by using “survival” and “survminer” packages in R software. And the ROC curves were drawn by using “survival,” “survminer” and “timeROC” packages. Moreover, the distributions of risk scores and survival statuses were plotted by “pheatmap” package.

### 2.6. Principal Component Analysis (PCA)

PCA with nonlinear methods was used to detect sample-to-sample heterogeneity by using “Rtsne” and “ggplot2” packages. The performances of the risk model were verified in both TCGA and GEO databases.

### 2.7. Independent Prognostic Value of the Risk Model

Univariate and multivariate Cox regression analyses were performed to determine the independent prognostic values of risk score and clinical factors (Age, Gender, Grade, Stage, and T, N, and M stages) using “survival” package. Clinical data were retrieved from both TCGA and GEO databases. Moreover, a heatmap was used to evaluate the differences if prognostic pyroptosis genes and risk scores in clinicopathological parameters by using “pheatmap” R package.

### 2.8. Gene Enrichment and Functional Annotation Analysis

Firstly, differentially expressed genes between high risk and low risk groups were identified by using “limma” package. Gene functional enrichment analyses were explored using Gene Ontology (GO) and Kyoto Encyclopedia of Genes and Genomes (KEGG) enrichment analysis. GO enrichment analysis was carried out using “clusterProfiler”, “org.Hs.eg.db,” “enrichplot,” and “ggplot2” R packages. KEGG analysis was developed using “clusterProfiler”, “org.Hs.eg.db,” “enrichplot,” and “ggplot2” R packages.

### 2.9. Immune Cells' Infiltration and Immune Function

CIBERSORT algorithm was used to estimate the abundances of various immune cell subsets in each sample. ESTIMATE method was used to calculate the immune scores, stromal scores, and estimate scores in TCGA-HNSCC cohort. Kaplan–Meier survival analysis was used to predict the prognostic values of various immune cells and immune functions. Immune cells' infiltration in different risk groups was explored using the single-sample Gene Set Enrichment Analysis (ssGSEA) based on the gene expression in TCGA-HNSCC cohort and GEO database. There were 16 types of immune cells and 13 types of immune functions being compared between high risk and low risk groups using “reshape” and “ggpubr” packages.

### 2.10. Calculation of the Tumor Mutation Burden

The somatic mutation data of HNSCC patients was downloaded and retrieved from the TCGA database. HNSCC patients were divided into high TMB and low TMB groups based on the median counts of TMB. Kaplan–Meier survival analysis was used to compare the survival rate between different groups by using “survival” and “survminer” packages in R software.

### 2.11. Tumor Immune Dysfunction and Exclusion (TIDE) Analysis

Data of tumor immune dysfunction and exclusion (TIDE) was downloaded from http://tide.dfci.harvard.edu/. TIDE could be used to validate the performance on predicting anti-PD1 and anti-CTLA4 responses. The correlations of the risk model with the dysfunction of tumor infiltrating cytotoxic T cells, exclusion of cytotoxic T cells by immunosuppressive factors, microsatellite instability (MSI), and TIDE prediction scores were assessed using “limma” and “ggpubr” packages in R.

### 2.12. Construction and Validation of a Nomogram Model

A nomogram model was constructed to comprehensively assess the survival probability of HNSCC patients, incorporating clinical factors (Age, Gender, Grade, Stage, and T, N, and M stages) and risk score. The predictive probabilities for 1-, 3-, and 5-year clinical outcomes were depicted by calibration curves. The sensitivity and specificity of the nomogram model in predicting 1-, 3-, and 5-year survival were determined using ROC curves. Moreover, the performance of the risk model in predicting OS was compared with TIDE scores and TIS signature using ROC curves.

Here, TIS signature is an 18-gene signature (CD3D, IDO1, CIITA, CD3E, CCL5, GZMK, CD2, HLA-DRA, CXCL13, IL2RG, NKG7, HLA-E, CXCR6, LAG3, TAGAP, CXCL10, STAT1, and GZMB) that correlated with the response to ICIs in malignancies [21, 22].

### 2.13. Statistical Analysis

All the data procession and statistical analyses were performed by using Perl software and R programming language (version 4.1.0). In all data analyses, a two-tailed *P* < 0.05 was considered statistically significant.

## 3. Results

### 3.1. Identification of Pyroptosis-Related Genes

**Figure 1:**
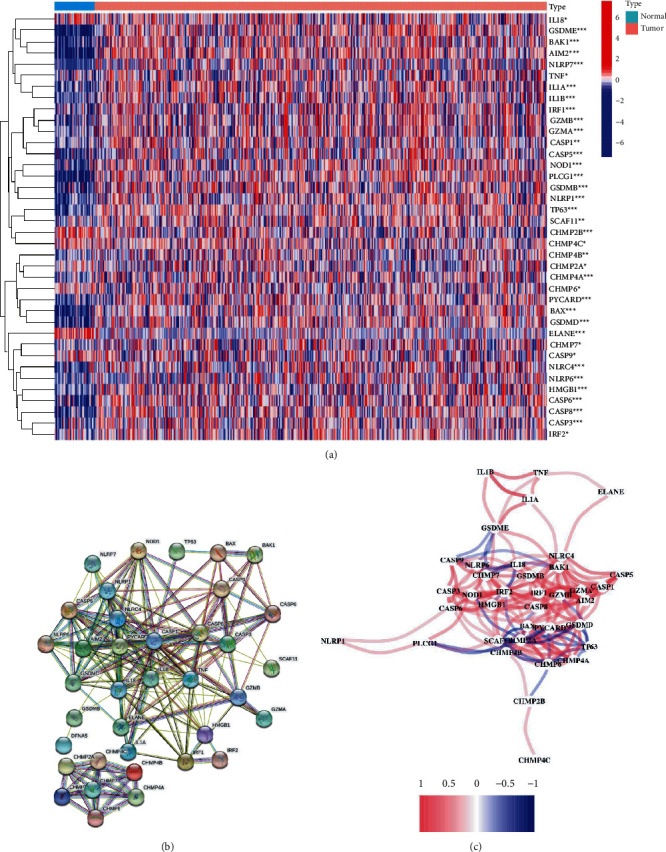
Identification of pyroptosis-related genes in HNSCC. Data were retrieved from the TCGA-HNSC database. (a) A heatmap of differentially expressed pyroptosis-related genes between normal tissues and HNSCC tumor tissues. (b) PPI network shows the interactions of the differentially expressed pyroptosis-related genes. (c) The correlation network of the differentially expressed pyroptosis genes. Red line represents positive correlation, while blue line represents negative correlation.

Firstly, the expression of 52 pyroptosis-related genes were retrieved from the TCGA-HNSCC cohort. The expressions of these 52 pyroptosis-related genes were compared between 44 normal tissues and 502 HNSCC tumor tissues. As shown in Figure 1(a), there were 38 pyroptosis-related genes with significant differences between normal tissues and tumor tissues (*P* < 0.05). Seven pyroptosis-related genes (ELANE, IL18, CHMP2B, CHMP4C, CASP9, CHMP6, and CHMP2A) were downregulated in tumor tissues, while 31 pyroptosis-related genes were upregulated in tumor tissues (Figure 1(a)). The interactions of these pyroptosis-related genes were analyzed by protein-protein interaction (PPI) network according to STRING database (Figure 1(b)). Moreover, the coexpressed network of these pyroptosis genes are visualized in Figure 1(c).

### 3.2. Patients Classification and Correlations with Clinical Parameters

HNSCC patients were classified using the consensus clustering analysis based on the differentially expressed pyroptosis-related genes. As shown in Supplementary Figure 1(a), when the *k*-value was 3, the HNSCC patients could be well classified. Kaplan–Meier survival analysis revealed that there were significant differences in the overall survival of HNSCC patients (*P*=0.002, Supplementary Figure 1(b)). Cluster 1 demonstrated better OS than cluster 2 and cluster 3, while cluster 3 exhibited poorer OS than cluster 1 and cluster 2. Moreover, the correlations between the gene expression profiles and variable clinical parameters (Age, Gender, Grade, Stage, and T, N, and M stages) were further studied. The results were depicted in a heatmap, where there were significant differences in Grade (*P* < 0.05) and Gender (*P* < 0.05) between different clusters (Supplementary Figure 1(c)).

### 3.3. Construction and Validation of a Pyroptosis-Related Prognostic Signature

**Figure 2:**
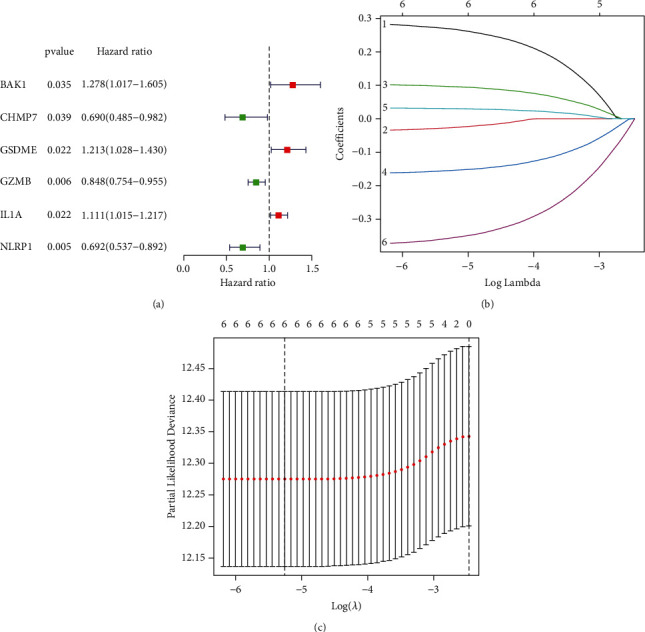
Construction of a pyroptosis-related risk model. (a) A forest map depicting 6 differentially expressed pyroptosis genes identified by univariate Cox regression analysis. (b and c) LASSO regressing analysis of hub pyroptosis-related genes. (b) LASSO coefficient profiles of the 6 prognostic pyroptosis genes. (c) Cross-validation for tuning the parameter in the LASSO regression.

We next constructed a prognostic signature by using univariate Cox regression analysis and LASSO analysis. As shown in Figure 2(a), there were 6 genes that demonstrated prognostic values according to univariate Cox regression analysis (*P* < 0.05). Here, BAK1 (*P*=0.035, HR: 1.278 (1.017–1.605)), GSDME (*P*=0.022, HR: 1.213 (1.028–1.430)), and IL1A (*P*=0.022, HR: 1.111 (1.015–1.217)) were poor prognostic pyroptosis-related genes, while CHMP7 (*P*=0.039, HR: 0.690 (0.485–0.982)), GZMB (*P*=0.006, HR: 0.848 (0.754–0.955)), and NLRP1 (*P*=0.005, HR: 0.692 (0.537–0.892)) were good prognostic pyroptosis-related genes.

LASSO regression analysis was performed to verify the risk model. The corresponding LASSO coefficient profiles and a partial likelihood deviation plot are shown in Figures 2(b) and 2(c), respectively.

### 3.4. Validation of the Pyroptosis-Related Gene Prognostic Signature

**Figure 3:**
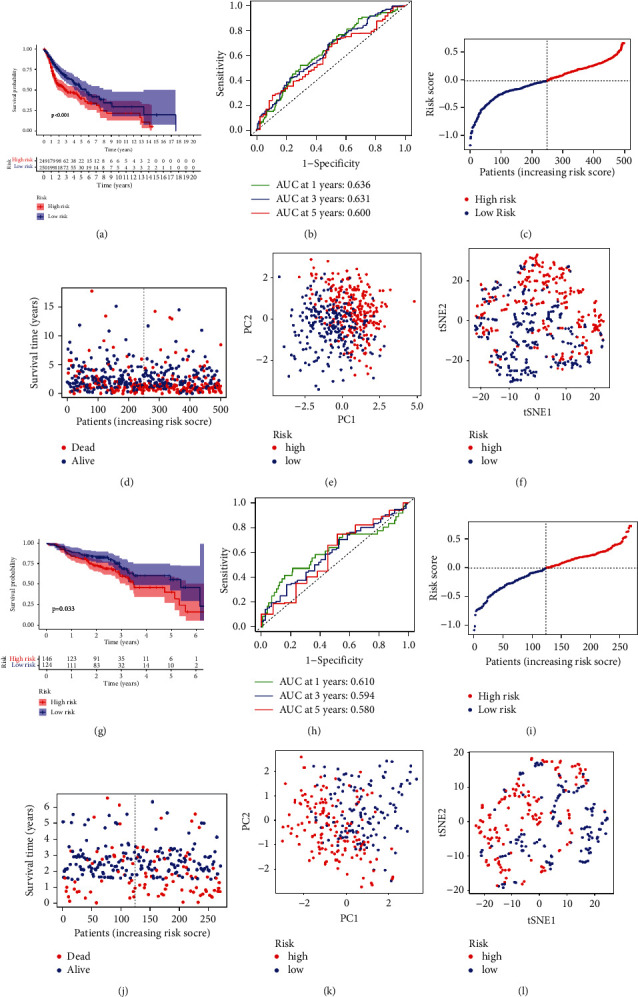
Performance of the risk model based on TCGA database. RNA-sequencing data and corresponding clinical data were downloaded from the TCGA database (a–f) and GEO database (g–l). (a) Kaplan–Meier survival analysis showed that the overall survival time of high risk group was shorter than that of low risk group. (b) The ROC curve of measuring the predictive value of the risk model. (c) Risk score distributions of each patient. (d) Survival status distribution of each patient. (e) PCA could separate pyroptosis-related genes in TCGA database. (F) tSNE results of the risk scores. (g) The overall survival time of high risk group was shorter than the low risk group based on Kaplan–Meier survival analysis. (h) Time-dependent ROC curves for 1-year, 3-year, and 5-year survival. (i) Distribution of risk score of HNSCC. (j) Survival status of each patient. (k) PCA plot for risk scores. (l) tSNE results of the risk scores.

HNSCC patients were divided into high risk and low risk groups based on the median value of the risk scores. Survival data were retrieved from both TCGA and GEO databases. TCGA-HNSCC data showed that patients in high risk group demonstrated poorer overall survival than low risk group (Figure 3(a), *P* < 0.001). The sensitivity and specificity of the prognostic model were evaluated by time-dependent receiver operating characteristic (ROC) analysis and principal component analysis (PCA). As shown in Figure 3(b), the area under curve (AUC) values were 0.636 for 1-year, 0.631 for 3-year, and 0.600 for 5-year survival, respectively. The risk scores distribution and survival status distribution between high risk and low risk groups are shown in Figures 3(c) and 3(d). The principal component analysis (PCA) results also demonstrated that the patients could be well classified by the risk scores (Figures 3(e) and 3(f)).

GEO data also confirmed the prognostic significance of the signature. As shown in Figure 3(g), there was significant difference in the overall survival time between the high risk and low risk groups (Figure 3(g), *P* < 0.001). Moreover, the AUC of time-dependent ROC curves at 1, 3, and 5 years were 0.610, 0.594, and 0.580, respectively (Figure 3(h)). And the distributions of risk scores, survival status, and PCA results of the risk model are shown in Figures 3(i)–3(l).

### 3.5. Prognostic Significance of the Risk Model

**Figure 4:**
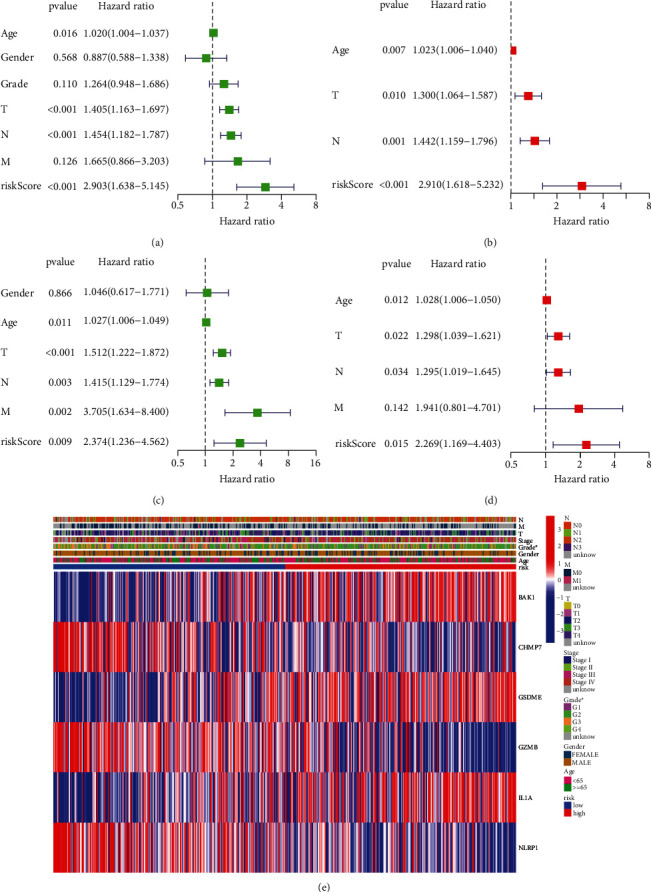
Independent prognostic value of risk model. (a) Univariate and (b) multivariate Cox regression analyses in TCGA cohort. (c) Univariate and (d) multivariate Cox regression analyses in GEO cohort. (e) A heatmap showed significant differences in Age, Gender, Grade, Stage, and T, N, and M stages and expression of prognostic pyroptosis-related genes between high risk and low risk groups.

Moreover, the prognostic significance of the risk score was assessed using univariate and multivariate Cox regression analyses. Data retrieved from the TCGA database showed that Age (*P*=0.007), T (*P*=0.010), N (*P*=0.001), and risk score (*P* < 0.001) were independent prognostic factors for HNSCC patients (Figures 4(a) and 4(b)). Data retrieved from GEO database also verified that Age (*P*=0.012), T (*P*=0.022), N (*P*=0.034), and risk score (*P*=0.015) were independent prognostic factors (Figures 4(c) and 4(d)).

Moreover, a heatmap depicted the correlations between the pyroptosis-related genes and risk scores. As shown in Figure 4(e), BAK1, GSDME, and IL1A were high-risk genes, while CHMP7, GZMB, and NLRP1 were low-risk genes. Moreover, there were significant differences in Grade between high risk and low risk groups.

### 3.6. Functional Enrichment Analysis of the Pyroptosis Gene Signature

We next explored the biological functions and signaling pathways underlying the risk model by using Gene Ontology (GO) enrichment analysis and Kyoto.

**Figure 5:**
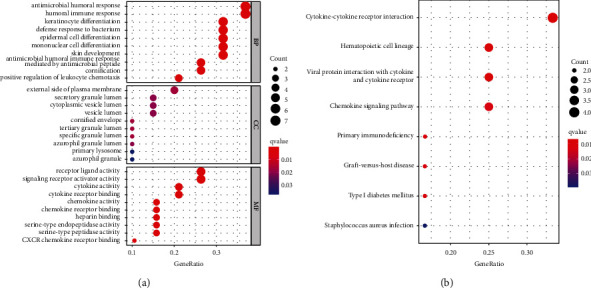
Functional enrichment analysis of the risk model. (a) GO enrichment analysis of the differentially expressed pyroptosis related genes between high risk and low risk groups. (b) KEGG enrichment analysis of the risk model.

*Encyclopedia of Genes and Genomes (KEGG) Pathway Analyses.* To begin with these studies, we firstly extracted 20 differentially expressed genes between high risk and low risk groups based on the TCGA database. GO enrichment analysis consisted of biological processes (BPs), molecular functions (MFs), and cellular components (CCs). GO results revealed that the functions of risk model were immune related (Figure 5(a)). KEGG results showed that the underlying signaling pathways included cytokine-cytokine receptor interaction and chemokine signaling pathway, and so on (Figure 5(b)).

### 3.7. Immune Landscape of HNSCC

**Figure 6:**
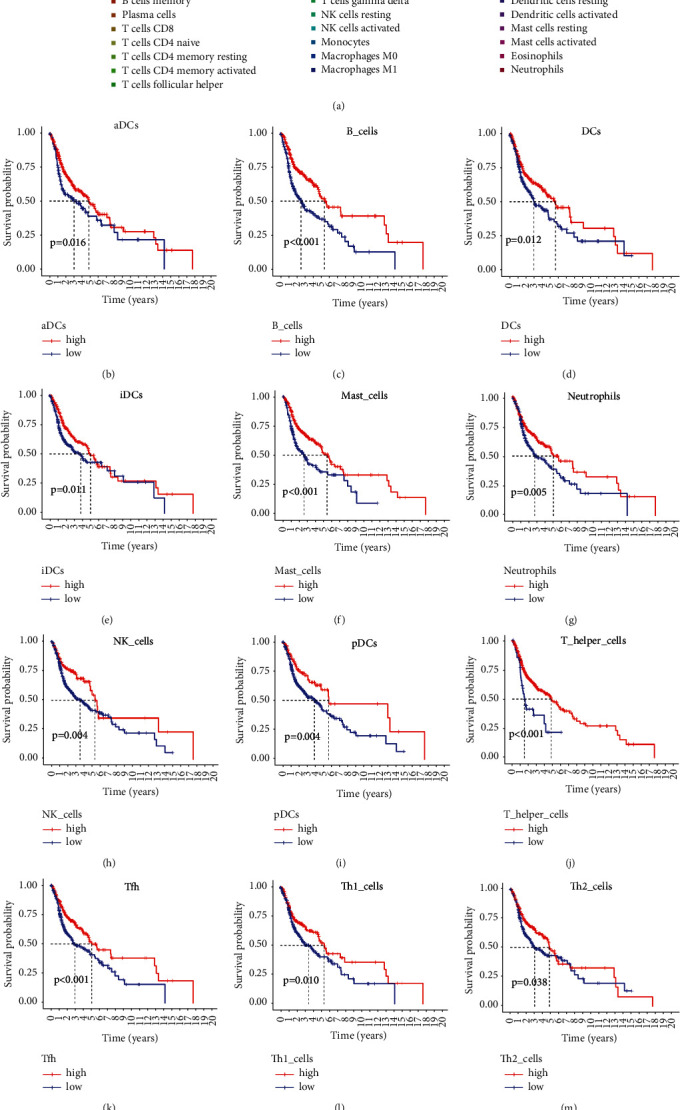
Immune cells infiltrating of HNSCC tumor immune microenvironment. Data were downloaded from the TCGA database. (a) A barplot shows the abundances of various immune cells in each sample. Kaplan–Meier curves of prognostic immune cells. OS curves for aDC (b), B cells (c), DCs (d), iDCs (e), Mast cells (f), neutrophils (g), NK cells (h), pDCs (I), T-helper cells (j), Tfh cells (k), Th1 cells (l), Th2 cells (m), TIL (n), Treg (o), and macrophages (p).

The immune landscape of HNSCC was analyzed using CIBERSORT algorithm. As shown in Figure 6(a), a barplot showed the infiltration of various immune cells. Next, the correlations between infiltrating immune cells and overall survival of HNSCC patients were analyzed by Kaplan–Meier survival curve. The high infiltration of aDC (Figure 6(b)), B cells (Figure 6(c)), DCs (Figure 6(d)), iDCs (Figure 6(e)), Mast cells (Figure 6(f)), neutrophils (Figure 6(g)), NK cells (Figure 6(h)), pDCs (Figure 6(i)), T helper cells (Figure 6(j)), Tfh cells (Figure 6(k)), Th1 cells (Figure 6(l)), Th2 cells (Figure 6(m)), TIL (Figure 6(n)), and Treg (Figure 6(o)) was positively correlated with better OS, whereas the high infiltration of macrophages could predict poor OS (Figure 6(p)).

**Figure 7:**
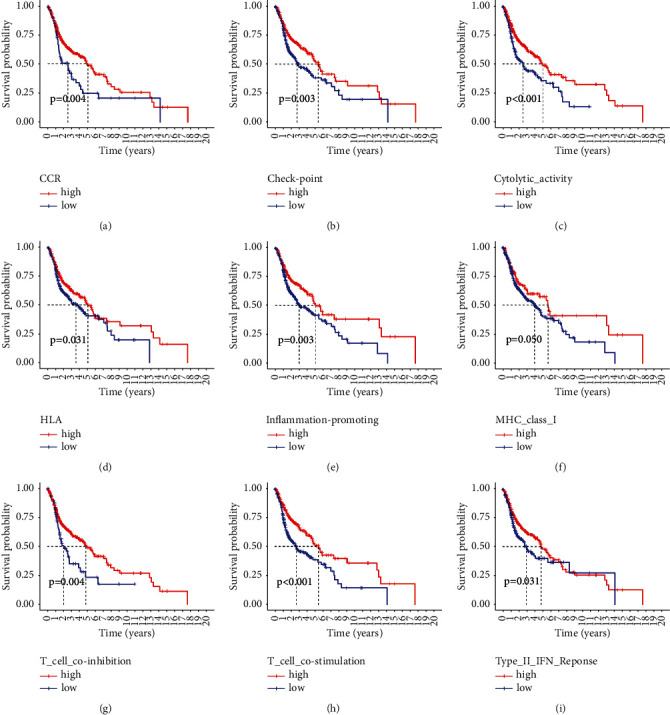
Immune functions of HNSCC tumor immune microenvironment. Data were downloaded from the TCGA database. High activity of CR (a), checkpoint (b), cytolytic activity (c), HLA (d), inflammation-promoting (e), MHC class I (f), T cells' coinhibition (g), T cells' costimulation (h), and type II IFN response (i) were positively correlated with OS of HNSCC patients.

We next studied the prognostic significances of immune functions in HNSCC. As shown in Figure 7, high activities of CCR (Figure 7(a), *P*=0.004), checkpoint (Figure 7(b), *P*=0.003), cytolytic activity (Figure 7(c), *P* < 0.001), HLA (Figure 7(d), *P*=0.031), inflammation-promoting (Figure 7(e), *P*=0.003), MHC class I (Figure 7(f), *P*=0.050), T cells' coinhibition (Figure 7(g), *P*=0.004), T cells' costimulation (Figure 7(h), *P* < 0.001), and type II IFN response (Figure 7(i), *P*=0.031) were significantly correlated with better OS of cancer patients.

### 3.8. Immune Significance of the Pyroptosis Gene Signature

**Figure 8:**
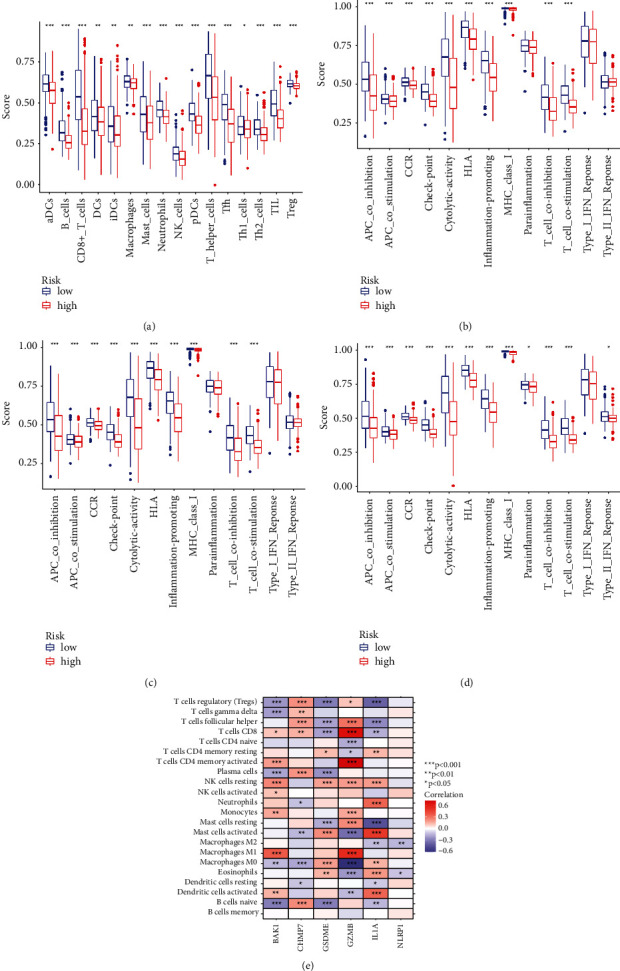
Correlations between risk score and the tumor immune microenvironment characterization of HNSCC. (a) Comparison of the enrichment scores of 16 types of immune cells between high risk group and low risk group in the TCGA-HNSC cohort. (b) Comparison of the enrichment scores of 13 immune-related pathways between high risk group and low risk group in the TCGA-HNSC cohort. (c and d) Comparison of immune cells infiltration and immune functions between high risk group and low risk group in GEO database. (e) Correlations between the risk genes and various immune cells.

Single-sample gene set enrichment analysis (ssGSEA) was employed to assess tumor immune-cells' infiltration and immune functions between high risk and low risk groups in both TCGA and GEO databases. In the TCGA-HNSCC cohort, high risk group demonstrated generally lower levels of 16 types of immune cells (Figure 8(a)). Meanwhile, high risk group also demonstrated lower activity of 10 kinds of immune functions, such as APC coinhibition, APC costimulation, CCR (chemokine and chemokine receptor), checkpoint, and so on (Figure 8(b)). Similar results were generated from the GEO database. The infiltration of 16 types of immune cells and 11 kinds of immune functions was lower in high risk group than that in low risk group (Figures 8(c) and 8(d)).

Moreover, the correlations between the prognostic pyroptosis-related genes and various immune cells are summarized in Figure 8(e). Here, red color represents positive correlation, while blue color represents negative correlation.

### 3.9. Significance of Pyroptosis-Related Gene Signature in Immunotherapy

We next studied whether pyroptosis-related gene signature could contribute to clinical benefit of immune checkpoint inhibitor treatment. TIDE analysis showed that there were no significant differences in dysfunction of tumor-infiltrating cytotoxic T cells (A), exclusion of cytotoxic T cells by immunosuppressive factors (B), microsatellite instability (MSI) (C), and TIDE prediction scores (D) between high and low risk groups (Supplementary Figure 2).

### 3.10. Prognostic Values of the Risk Model Combined with the TMB

**Figure 9:**
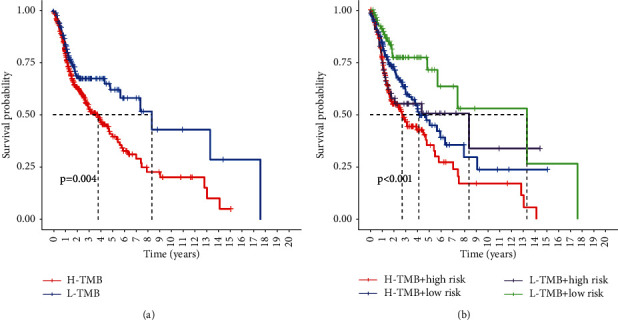
The prognostic significance of TMB combined risk score in HNSCC patients. (a) Kaplan–Meier survival analysis of the TMB in predicting the survival of HNSCC patients. (b) Kaplan–Meier survival analysis showed the prognostic values of TMB combined risk score.

After calculating the TMB values, the HNSCC patients were classified into high-TMB and low-TMB groups based on the median value of TMB. Kaplan–Meier (K-M) survival analysis with log-rank tests indicated that the low-TMB group demonstrated better OS than high-TMB group (Figure 9(a), *P*=0.004).

We next combined the risk score and TMB to predict the survival of HNSCC patients. As shown in Figure 9(b), high TMB and high risk predicted the worst OS, while low-TMB and low risk group demonstrated the best prognosis. These results indicated that risk score and TMB could be utilized simultaneously for predicting patient prognoses.

### 3.11. Construction of a Prediction Nomogram

**Figure 10:**
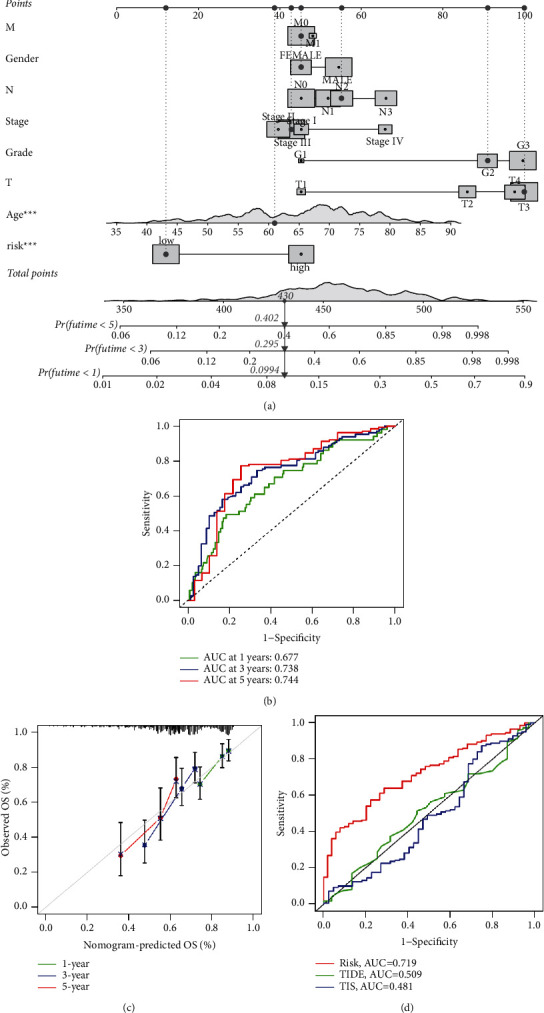
Construction of a nomogram. (a) Nomogram predicting the OS of HNSCC patients. (b) Time-dependent ROC curves for the nomogram in predicting the OS. (c) The calibration curves for predicting patients' OS at 1, 3, and 5 years. (d) Time-dependent ROC curves for risk model. TIDE scores and TIS signature in predicting the OS of HNSCC patients.

Moreover, a nomogram was constructed for predicting the prognosis of HNSCC patients by combining the 6 pyroptosis-related gene signature and conventional clinical parameters, including Age, Gender, Grade, Stage, and T, N, and M stages (Figure 10(a)). The performance of the nomogram was assessed using AUC index of ROC analysis and calibration curve. The AUCs of nomogram model for predicting 1-, 3-, and 5-year overall survival rates were 0.677, 0.738, and 0.744, respectively (Figure 10(b)). Calibration curve for the probability of 1-, 3-, and 5-year OS demonstrated good consistency between the actual observed survival and the nomogram-based prediction (Figure 10(c)). What is more, the predictive capabilities of risk score, TIDE, and TIS were 0.719, 0.509, and 0.481, respectively, indicating that the pyroptosis-related gene signature has better sensitivity and specificity in predicting the OS of HNSCC patients (Figure 10(d)).

## 4. Discussion

Pyroptosis was reported to be involved in the tumor progression and demonstrated great potential in cancer treatment [23]. As a form of programmed necrotic cell death, pyroptosis was like a double-edged sword in malignancies [10–12]. On the one hand, pyroptosis could kill the tumor cells and mediate tumor regression [10, 11]. On the other hand, pyroptosis could modify the tumor microenvironment to promote tumor growth [12]. The effects of pyroptosis on the biological functions of tumor cells depended on various pyroptosis-related genes. Moreover, the expression of pyroptosis-related genes were correlated with the survival of some cancer patients, including ovarian cancer [24], lung adenocarcinoma [25], skin cutaneous melanoma [20], gastric cancer [26], and laryngeal squamous cell carcinoma [27]. However, the expression and prognostic values of pyroptosis-related genes in HNSCC still need to be comprehensively elucidated.

Here, a list of pyroptosis-related genes was retrieved by literature searching, which includes 52 pyroptosis-related genes. And thirty-eight out of 52 pyroptosis-related genes were differentially expressed between normal tissues and HNSCC tumor tissues. Three clusters, divided by consensus clustering analysis, demonstrated significant differences in the OS of HNSCC patients, as well as other clinical factors including gender and grade. What is more, univariate Cox regression analysis and LASSO Cox analysis were performed to construct a pyroptosis-related gene prognostic signature. The risk model was consisted of 6 pyroptosis-related genes, including BAK1, GSDME, IL1A, CHMP7, GZMB, and NLRP1. Most of these six prognostic pyroptosis-related genes were once reported to be correlated with the progression and prognosis of cancer patients. BAX1, also known as autophagy-related gene and apoptosis signaling molecule, could predict the survival of head and neck cancer [28–30]. Pan-cancer analysis revealed that GSDME was highly expressed in most malignancies and significantly correlated with patients' survival [31]. Ibrahim et al. identified GSDME as a promising biomarker in the detection of colorectal cancer [32]. IL1A, as a proinflammatory cytokine, was upregulated in many types of cancers, such as breast cancer and lung cancer [33, 34]. Induction of IL1A could promote proliferation, angiogenesis, and metastasis of tumor cells [33]. You et al. found that abnormal IL1A expression was correlated with poor prognosis of triple negative breast cancer [35]. CHMP7, as a ESCRT-III-related protein, was related to nuclear envelope reformation [36]. CHMP7 was identified as a prognostic biomarker in gastric cancer [37]. Granzyme B (GZMB), as an inflammatory gene and cytotoxic gene, participated in immune response and tumor cell killing. GZMB was identified as a progression biomarker in basal-like breast cancer [38]. NLR family, pyrin domain containing 1 (NLRPL) could form inflammasome and was involved in immune responses and cell death [39]. Low expression of NLRP1 was correlated with poor overall survival in lung adenocarcinoma [25, 40]. The expression and prognostic significance of most of these prognostic pyroptosis-related genes have never been reported in HNSCC. In this study, all these genes were significantly correlated with the OS of HNSCC patients. Multiple genes risk model usually performed better sensitivity and specificity than single gene in predicting OS, as single gene usually showed lower sensitivity and specificity based on ROC analysis. Here, our risk model demonstrated good performance according to data generated from TCGA and GEO database. High risk group had a significantly poorer overall survival than the low risk group. Univariate and multivariate Cox regression analyses further verified that the risk score could be used as an independent prognostic factor for HNSCC patients.

We next explored the potential functional enrichment of the pyroptosis-related gene signature. GO and KEGG analyses indicated that the risk model was mainly involved in immune responses, cytokine activity, and chemokine and chemokine receptor activity. These results strongly indicated that pyroptosis exhibited a significant correlation with immune microenvironment. CIBERSORT estimation results showed the abundance of immune cells infiltration and immune functions in the tumor microenvironment of HNSCC. Indeed, previous studies have indicated that there were close correlations between pyroptosis and immune response and immune cells infiltration. Pyroptosis could stimulate effective immune responses. Wang et al. found that nanoparticle-conjugated gasdermin could sensitize 4T1 tumor cells to anti-PD-1 therapy strategy [16]. The expression of Gasdermin E (GSDME) was linked with the cancer-associated fibroblast infiltration in multiple malignancies [31]. Increased expression of GZMB was connected with high numbers of mutations in non-small-cell lung cancer [41]. Diminished expression of GZMB was associated with higher PD-1, PD-L1, LAG-3, and TIM-3 expression in invasive bladder cancer [42]. The expression of NLRP1 was positively correlated with the degree of tumor-infiltrating immune cells in lung adenocarcinoma. Accordingly, both TCGA and GEO data revealed that there were significant differences in the immune cells infiltration and immune functions between the high risk and low risk groups. High risk group demonstrated decreased expression of immune cells and repressed activities of immune functions. All the prognostic pyroptosis-related genes were significantly correlated with the infiltration of both positive and negative immune cells, such as Tregs, macrophage M0, macrophage M1, macrophage M2, CD8+T cells, and so on. All these data strongly indicated that our pyroptosis related risk model was associated with the tumor immune microenvironment. However, there were no significant differences in tumor immune dysfunction and exclusion between high and low risk groups. Even ICIs targeting PD-1, such as Nivolumab and Pembrolizumab, have been approved in the treatment of HNSCC [4, 43]. About 70% of HNSCC patients failed to benefit from ICIs [43]. Our data partially explained the low response rate to ICI in HNSCC. What is more, the immune-related mechanisms underlying pyroptosis might not be PD-1/PD-L1 or CTLA-4 related. There must be other immune-related mechanisms that needed to be further elucidated. Finally, a novel HNSCC nomogram was constructed according to the risk score and clinical parameters. AUC values and calibration curves demonstrated good predictive ability. Meanwhile, the sensitivity and specificity of the risk model were much better than TIDE score and TIS signature.

Intensive studies have shown that HNSCC exhibited enormous tumor heterogeneity, leading to different clinicopathological characters and therapeutic responses based on different tumor regions, distinct mutational profiles, and variable molecular characters [44]. The main limitation of this study was that we constructed the pyroptosis gene signature to predict OS and immune response incorporated all HNSCC patients without considering tumor heterogeneity. It is worthwhile to further explore the relationships between pyroptosis genes and various phenotypes of HNSCC, exploring their potential clinical applications to target different types of HNSCC patients.

## 5. Conclusion

In conclusion, our study provided a novel model for predicting the survival of HNSCC. This model showed good correlations with tumor immune microenvironment.

## Data Availability

All data in our study are available from the corresponding authors upon reasonable request.

## Abbreviations

HNSCC: Head and neck squamous cell carcinoma
GEO: Gene Expression Omnibus
TMB: Tumor mutation burden
LASSO: Least absolute shrinkage and selection operator
ROC: Receiver operating characteristic
PCA: Principal component analysis
GO: Gene Ontology
KEGG: Kyoto Encyclopedia of Genes and Genomes
OS: Overall survival
ssGSEA: Single-sample gene set enrichment analysis
TIDE: Tumor immune dysfunction and exclusion
TIS: T-cell-inflamed signature
ICI: Immune checkpoint inhibitor
PPI: Protein-protein interaction
STRING: Search tool for recurring instances of neighbouring genes
AUC: Area under the curve
BPs: Biological processes
MFs: Molecular functions
CCs: Cellular components
MSI: Microsatellite instability
GZMB: Granzyme B
GSDME: Gasdermin E.

## Supplementary Materials

Supplementary Materials
*Supplementary Figure 1.* Consensus clustering analysis of HNSCC patients according to differentially expressed pyroptosis-related genes. (A) Heatmap of sample consensus after setting *k* = 3. (B) Kaplan–Meier survival analysis showed the differences in the overall survival between different clusters. (C) A heatmap of the three clusters along with clinicopathological factors. *Supplementary Figure 2.* TIDE analysis of the risk model. Associations of the risk score with dysfunction of tumor infiltrating cytotoxic T cells (A), exclusion of cytotoxic T cells by immunosuppressive factors (B), microsatellite instability (MSI) (C), and TIDE prediction scores (D).
