# Supplementary material for: Prognosis and Characterization of Immune Microenvironment in Head and Neck Squamous Cell Carcinoma through a Pyroptosis-Related Signature
Source: J Oncol. 2022 Apr 6;2022:1539659. doi: 10.1155/2022/1539659 (PMC9007648; doi:10.1155/2022/1539659)
Supplement: Supplementary Materials — Supplementary Figure 1. Consensus clustering analysis of HNSCC patients according to differentially expressed pyroptosis-related genes. (A) Heatmap of sample consensus after setting k = 3. (B) Kaplan–Meier survival analysis showed the differences in the overall survival between different clusters. (C) A heatmap of the three clusters along with clinicopathological factors. Supplementary Figure 2. TIDE analysis of the risk model. Associations of the risk score with dysfunction of tumor infiltrating cytotoxic T cells (A), exclusion of cytotoxic T cells by immunosuppressive factors (B), microsatellite instability (MSI) (C), and TIDE prediction scores (D). [file 1539659.f1.docx]

**Supplementary material**

**
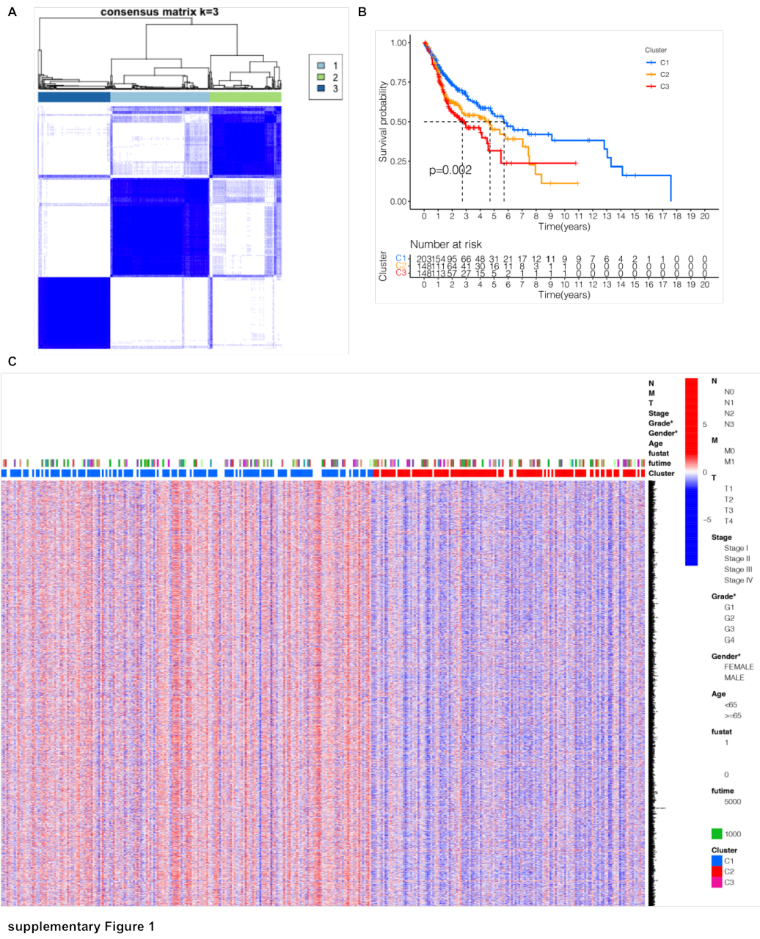
**

**Supplementary Figure 1. Consensus clustering analysis of HNSCC patients according to differentially expressed pyroptosis-related genes.** (A) Heatmap of sample consensus after setting k =3. (B) Kaplan Meier survival analysis showed the differences in the overall survival between different clusters. (C) A heatmap of the three clusters along with clinicopathological factors.


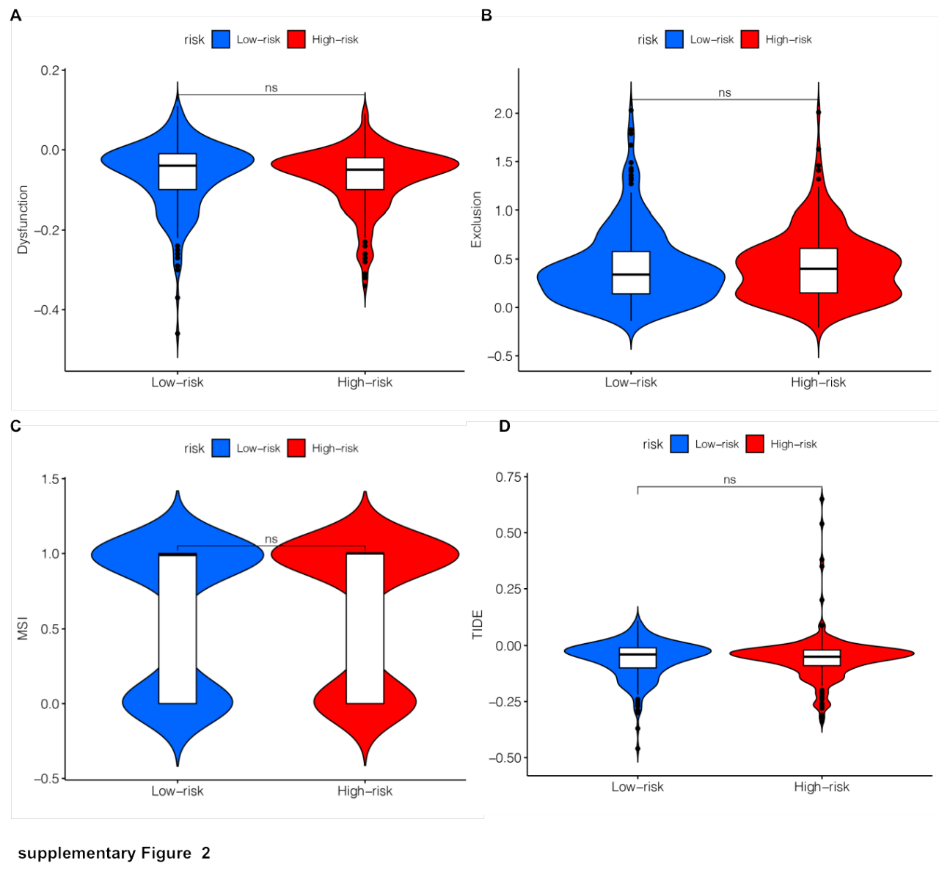


**Supplementary Figure 2.** TIDE analysis of the risk model. Associations of the risk score with dysfunction of tumor infiltrating cytotoxic T cells (A), exclusion of cytotoxic T cells by immunosuprresive factors (B), microsatellite instability (MSI) (C) and TIDE prediction scores (D).
